# Uptake and engagement with digital mental health in the workplace: A mixed-methods analysis of the EMPOWER trial

**DOI:** 10.1016/j.invent.2026.100911

**Published:** 2026-01-20

**Authors:** Stijn B. Peeters, Marleen de Mul, Frederick W. Thielen, Marjo Sinokki, Kaja Staszewska, Luis Salvador-Carulla, Sue Lukersmith, Beatriz Olaya, Leona Hakkaart-van Roijen

**Affiliations:** a Erasmus school of Health Policy & Management, Erasmus University Rotterdam, Rotterdam, the Netherlands; b Länsirannikon Työterveys, Turku, Finland; c Unit of Public Health, University of Turku, Turku, Finland; d Nofer Institute of Occupational Medicine, Lodz, Poland; e Health Research Institute, Faculty of Health, University of Canberra, ACT, Australia; f Research, Innovation and Teaching Unit, Parc Sanitari Sant Joan de Déu, Universitat de Barcelona, Sant Boi de Llobregat, Spain; g Centro de Investigación Biomédica en Red de Salud Mental (CIBERSAM), Ministry of Science and Innovation, Madrid, Spain; h Department of Clinical and Health Psychology, Autonomous University of Barcelona, Bellaterra, Spain

**Keywords:** Digital health, Uptake, Engagement, Digital intervention, Occupational health, Workplace mental health

## Abstract

This study examined contextual factors influencing the uptake and use of the EMPOWER digital mental health platform, implemented in small and medium-sized enterprises and public agencies in Spain, Poland, Finland, and the United Kingdom. The platform was developed within an EU-funded project to promote workplace mental health and evaluated in a randomised controlled trial assessing its effectiveness and cost-effectiveness. A mixed-methods design was applied combining logistic regression analyses of baseline employee data with qualitative semi-structured interviews exploring barriers and facilitators to engagement. Results indicated that successful uptake was supported by strong employer involvement, a positive workplace culture, clear communication of benefits and data privacy, tailoring of content to employee needs, and available technical support. Barriers included insufficient communication, limited organisational support, lack of allocated time for use, unclear instructions, and concerns about anonymity. Employers often expressed reluctance to take responsibility for facilitating implementation, reflecting low organisational readiness. While the platform itself was generally regarded as user-friendly, its integration into daily workplace practices was inconsistent, with many employees using it outside of working hours. In conclusion, effective and sustainable implementation of digital workplace mental health interventions requires more active stakeholder engagement, clearer and sustained communication strategies, and alignment with organisational policies and structures. Addressing these contextual factors is essential for maximising uptake and ensuring that digital health platforms such as EMPOWER achiever their intended impact in supporting mental health at work.

## Highlights

• Successful uptake of workplace digital health interventions depends on strong employer engagement and supportive culture.
• Low organisational readiness and unclear stakeholder roles can significantly hinder uptake.
• Cultural differences shape employee engagement with mental health interventions, requiring context-specific strategies.

## 1 Introduction

The impact of mental health problems on the workforce is both far-reaching and significant. At any given moment, around one-sixth of the working-age population experiences mental disorders (OECD, 2012). These challenges deeply influence employee well-being, productivity, and absenteeism rates (Stratton et al., 2017). Interestingly, work is often considered both a contributing factor to mental health problems and a critical means of improving mental health, fostering social involvement and overall wellbeing of individuals (van Hees et al., 2022).

Even though mental health problems have now taken the forefront as the primary reason for work disability and absence, workplace mental health interventions have fallen behind (Stratton et al., 2017). As a result, there has been an emphasis towards promoting mental health in the workplace as a strategic priority (Leka and Nicholson, 2019). One of the interventions that has the potential to promote occupational mental health is digital health, defined as “the use of information and communication technologies (ICT) for health” (eHealth at WHO [Internet], 2018). Interventions based on digital health present the benefit of a reduced barrier to entry for participation, compared to face-to-face consultations with a therapist (Christie et al., 2018). It has shown to be effective in reducing and preventing overall mental health problems in two systematic reviews, both targeted at the general population (Deady et al., 2017) and employees (Stratton et al., 2017), providing proof to support the potential application as a low-cost intervention for employees.

However, most studies on digital health interventions in the workplace focus on physical health problems (Christie et al., 2021; Tran et al., 2017) (e.g., traumatic brain injury) or have been implemented in the healthcare sector only (Cremers et al., 2021; Boyne and Vrijhoef, 2013; Kujala et al., 2020) (e.g., mental health of nurses), leaving a gap in understanding the uptake of mental health-focused digital workplace interventions in other sectors. This is particularly important as only few digital health initiatives achieve their intended effects on patient-relevant outcomes when implemented in practice (Cremers et al., 2021). Their success often hinges on factors at the individual level (e.g., attitudes of intermediaries like employers) and organisational factors (e.g., strategic alignment or organisational support), which are critical to the effectiveness of implementation processes (Cremers et al., 2021).

The European Platform to Promote Wellbeing and Health in the workplace (EMPOWER) was aimed at developing a comprehensive and integrated digital health platform, and app, designed to enhance overall well-being and reduce the negative effects of mental health problems within the workplace (Olaya et al., 2022). The EMPOWER platform was developed within an EU-funded research project and implemented in Small and Medium Enterprises (SMEs) and public agencies in Spain, Poland, Finland, and United Kingdom as part of a Randomized Controlled Trial (RCT) to assess (cost-) effectiveness. To complement this evaluation, the present study combines qualitative and quantitative approaches to examine factors influencing the uptake and use of the EMPOWER platform.

Specifically, this study addresses two overarching objectives. First we explore perceptions of workplace mental health and identify individual, organisational, and contextual factors that facilitate or hinder the uptake and use of a digital mental health intervention. Second, we examine which user characteristics and workplace-related factors are associated with subsequent use of the EMPOWER application. By integrating qualitative insights with quantitative findings and the results of the (cost-)effectiveness analysis, this study aims to improve the interpretation of economic and effect outcomes of EMPOWER, explain variation in engagement, and inform strategies to enhance the implementation of digital mental health interventions in diverse workplace settings.

## 2 Methods

### 2.1 Design and setting

This study on the use and uptake of the EMPOWER intervention is part of a larger international project evaluating a workplace-based digital health platform aimed at promoting mental health and wellbeing and reducing the negative consequences of mental health problems at work. The overall aim and methodology of the EMPOWER trial have been described in detail elsewhere (Olaya et al., 2022; Van Der Feltz-Cornelis et al., 2023). Briefly, EMPOWER is a multi-modal, self-guided intervention that combines universal (primary), targeted (secondary), and tertiary prevention strategies, addressing mental health awareness, psychosocial risks at work, wellbeing, early detection of mental health problems, healthy behaviours, and work functioning.

The trial was conducted among employees from SMEs and public agencies of four countries: Finland, Poland, Spain and the United Kingdom (UK). To be eligible for participation, individuals were required to meet the following criteria: 1) be 18 years of age or older; 2) possess a mobile phone with internet access; 3) have an adequate understanding of the local language; and 4) have provided informed consent. Participants were invited by email through their employer and enrolled in the EMPOWER trial according to the original study protocol. Although access to the full mobile intervention differed in timing across participants, all participants eventually received access to the complete EMPOWER platform during the course of the trial.

The EMPOWER platform consisted of two interconnected components: a public website and a mobile application. The website provided general information and awareness-raising material on mental health in the workplace, including content related to stigma reduction and psychosocial working conditions. The mobile application constituted the core components of the intervention and included self-guided screening tools to identify stressful psychosocial working conditions, presenteeism, absenteeism, and mental and physical systems, as well as modular content aimed at promoting wellbeing, mental health, and work functioning.

This study followed a mixed-methods design incorporating quantitative and qualitative components. The quantitative component adhered to the original EMPOWER trial protocol (Olaya et al., 2022) and was based on baseline employee data, collected prior to participants' exposure to the full intervention, as well as on usage data from the same participants who had access to the EMPOWER application. These linked baseline and usage data were used to examine how individual characteristics were associated with later uptake and engagement.

The qualitative component complemented the (cost-)effectiveness analysis by providing in-depth insights into contextual factors influencing the uptake and use of the EMPOWER platform, helping to explain variations in engagement and economic outcomes that cannot be captured through quantitative cost and effect measures alone. The results of the trial in terms of effectiveness will be reported elsewhere.

In the current study, both the employee and employer perspectives were essential for understanding facilitators and barriers to effective uptake in the workplace. Hence, semi-structured, one-on-one online interviews were conducted with employers and employees at two distinct time points. Due to resources restrictions, the interviews were performed in three of the four countries participating in the trial: Finland, Poland and Spain. The baseline (T0) interviews were conducted within one week after completion of the quantitative baseline assessment, at a stage when these participants had access only to the general website content. The post-intervention (T1) interviews took place immediately after the completion of the T1 questionnaires (post-treatment assessment), approximately eight weeks after participants had gained access to and completed the full EMPOWER mobile intervention.

### 2.2 Participants and sampling

This study aimed to include the widest possible diversity of respondents from the original trial, employing different selection processes for the qualitative and quantitative analyses. A comprehensive description of the methodology used to establish the EMPOWER population is provided elsewhere (Olaya et al., 2022).

#### 2.2.1 Participants and sampling for the qualitative analysis

Employees and employers who did not receive immediate access to the full EMPOWER platform, but instead gained access at a later stage were approached for the qualitative study. This approach was chosen for two reasons: first, their later access to the full mobile application made it possible to conduct interviews both before and after app use; second, it increased the likelihood that major technical issues would have been identified and resolved before they began using the full intervention, allowing more reliable feedback on the platform's performance.

Employees from Poland, Spain and Finland, were contacted by local research teams and invited to participate in the qualitative interviews after providing consent and completing the baseline questionnaire. Employers were included based on their involvement in the organisations participating in the trial. Respondent selection followed the principles of purposive sampling, with a specific focus on the roles or professions of the participants. The aim was to include a large variety of respondents on the following characteristics: age group, type of employer (SME, public) or employee (white collar, blue collar), and gender. During the trial, the United Kingdom opted out of this qualitative study, due to constraints related to limitations of time and personnel.

From those willing to participate in the interviews, 40 employees were interviewed at baseline: 12 from Spain, 20 from Finland, and 8 from Poland. At follow-up (post-intervention), 16 employees were interviewed, 4 from Spain, 8 from Finland, and 8 from Poland. Most employees were female, with all participants in Poland being women. At baseline (T0), participants were predominantly higher educated, although this distinction became less pronounced by T1, as a result of dropout. Employment patterns also shifted: at T0, most respondents worked in public agencies, but by T1, the gap between those employed in public agencies and SMEs had narrowed. Further details can be found in Table 1 in Appendix A.

Among employers, 24 baseline interviews were conducted: 8 in Spain, 9 in Finland, and 7 in Poland. At follow-up, 12 interviews were conducted: 3 in Spain, 4 in Finland, and 5 in Poland. At T0, the majority of employers worked in public agencies. By T1, those willing to be interviewed were evenly distributed between SMEs and public agencies. Employer roles at T0 were nearly evenly divided among directors, managers, and principals. By T1, however, nearly all employers held managerial or principal positions. Characteristics of the employers, including company information, are further detailed in Appendix A, Table 3.

#### 2.2.2 Participants and sampling for the quantitative analysis

Quantitative data were based on all participants who completed the baseline questionnaire prior to receiving the full intervention, regardless of their allocation within the original EMPOWER trial. In addition, usage data were analysed for all participants who had access to and used the EMPOWER mobile application. The quantitative population primarily comprised employees from the United Kingdom, most of whom were female and employed in public institutions. A detailed breakdown of this population is presented in Table 2 of Appendix A.

### 2.3 Data collection

#### 2.3.1 Data collection for the qualitative analysis

The topic list for the baseline interviews covered the firsthand encounters of participants with the EMPOWER platform, involving their personal expectations, as well as the obstacles and factors that either impede or enhance its optimal utilisation. Questions for both the employers and employees were therefore based on the following topics: attitude towards mental health in the workplace; attitude towards the platform; usability; usefulness; impact; outcomes; and context of the implementation. The topic list for the post-intervention interviews followed the same structure but was more focused on the actual experience and the perceived impact of using the application.

The interviews were done by teleconferencing (audio taped Zoom meetings), by phone (the researcher took notes) or face-to-face, in the local language. After the interviews were conducted, the researcher responsible for the data collection transcribed the interviews and made a report in English with quotes. The reports were anonymous and did not contain any data that would allow the participant to be identified.

#### 2.3.2 Data collection for the quantitative analysis

Data for the quantitative part of the study were based on the EMPOWER baseline data, utilising descriptive, quality of life, productivity, and disease-specific questionnaires. In addition to the personal data collected at baseline, data regarding app usage was gathered. This application data consisted of the number of app launches, the programs (e.g., mood tracking, learning pills, stress prevention exercises, etc.) used within the application and the dates of application usage.

### 2.4 Data analysis

**Fig. 1:**
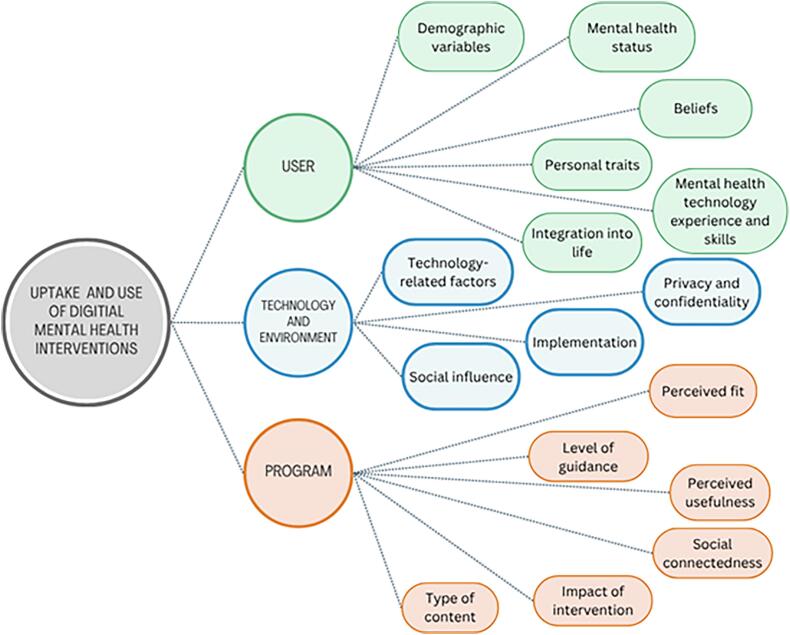
Conceptual model of the Uptake and Use of Digital Mental Health Interventions adapted from the publication of Borghouts et al. (Borghouts et al., 2021).

To define the areas of analysis, we drew on Borghouts et al. (Borghouts et al., 2021), who reviewed 208 studies on barriers and facilitators of engagement with digital mental health interventions. We assumed uptake and use depend on three constructs: user, technology & environment, and program. The user construct includes demographics, mental health status (symptom severity), personal traits, perceptions of mental and digital health, prior experience with technology and mental health, and ability to integrate interventions into daily life. The technology and environment construct covers privacy (anonymity), social influence (perceptions of peers, family, providers), and implementation (e.g., training). The program constructs highlights engagement when content is relevant and useful, supported by clear guidance, social connection, and positive effects (e.g., symptom reduction). These constructs are shown in Fig. 1.

Based on these key areas, we conducted thematic content analysis (Vaismoradi et al., 2013). Results were analysed and categorised for separately for baseline (T0) and post-intervention (T1) using Atlas.ti (Zabor et al., 2022). Initial coding was performed by one researcher and independently reviewed by another. Themes were compared across participant groups (employers vs. employees) and across countries to identify both shared and country-specific facilitators and barriers to uptake and use of the EMPOWER platform. Country was therefore used as an analytic dimension to examine whether themes emerged within or across national contexts. Observed country-specific patterns were described and interpreted in relation to their contextual setting, rather than formally compared across countries, as the study was not designed for cross-national comparison and sample composition differed between countries. As samples were independent, organisational overlap could not be determined. Results were mapped to themes, then coded as facilitators and barriers. To ensure transparency and protect participants' anonymity, quotations are presented using anonymised identifiers. Each quotation includes a participant ID (e.g., Employer 1 or Employee 1) and country indicated by a capital letter (S = Spain, F = Finland, P = Poland). For employees, identifiers additionally include gender, indicated by a lowercase f *(*female) or m *(*male), and age presented as a number. For employers, for whom gender and age were not collected, identifiers include participant ID and country only (e.g., Employee 12, F, *f*, 34 or Employer 3, P).

For the quantitative analysis a logistic regression model was used to examine the ‘user’ construct (Borghouts et al., 2021). Users were divided into active and inactive groups (Appendix C). A stepwise selection strategy (MASS in R (Venables and Ripley, 2002)) identified the model with the lowest AIC (Chowdhury and Turin, 2020). Categories which contained more than two categories, and had no hierarchical structure, were recoded into sum-to-zero contrasts. Multicollinearity was tested with VIF. Estimates and *p*-values were reported, with variables at *p* < 0.05 visualised. A linear regression with number of uses as the outcome validated the logistic regression. As the quantitative component included participants from all four countries (Finland, Poland, Spain, and the United Kingdom), with the United Kingdom representing the largest share of users, and the qualitative data were collected in Spain, Finland, and Poland only, findings from the two components were interpreted complementarily rather than triangulated at the country level.

### 2.5 Variables included in the analysis

Based on the stepwise variable selection and theoretical relevance, the following variables were included in the quantitative model: age, gender, education level, company size, PHQ-9, MHQoL, MHQoL-VAS, sitting time, iPCQ, and marital status. The selection process ensured that the final model had the best fit, with the lowest AIC value (304.8), and no VIF exceeded 5, indicating absence of multicollinearity (Kim, 2019).

The demographic variables included were measured as follows. Age was treated as a continuous variable, measured in years. Gender was categorised as male or female. Education level was classified as an ordinal variable, with levels ranging from primary school to a doctoral degree. Marital status was also a categorical variable, with categories such as single, cohabitating, and divorced. Lastly, company size was categorised based on the number of employees in participants' workplaces.

Mental health and well-being measures were assessed in this model using two key measures. The Patient Health Questionnaire (PHQ-9), evaluated the severity of depressive symptoms (Kroenke et al., 2001). Total scores ranged from 0 to 27 with severity classified into five categories: minimal (1–4), mild (5–9), moderate (10–14), moderately severe (15–19), and severe (20–27). The Mental Health Quality of Life (MHQoL) questionnaire measured mental well-being, where higher scores reflected better mental quality of life (mental QoL) (van Krugten et al., 2022). It also includes a visual analogue scale (MHQoL -VAS) ranging from 0 to 10.

Lifestyle and productivity-related variables were also incorporated. Sitting time was measured as a continuous variable, representing the number of hours per day spent in sedentary activities. Presenteeism was assessed using the iMTA Productivity Cost Questionnaire (iPCQ), where participants rated their work productivity on a scale from 0 to 10 (Bouwmans et al., 2015). This score was used to estimate the number of working hours during which individuals were not fully productive.

### 2.6 Ethics

The study is registered on Clinical Trials.gov under the identifier NCT04907604. The protocol for the qualitative assessment was approved by the ethics committee of Fundació Sant Joan de Déu (PIC-39-20), Institute of Occupational Medicine, University of Lodz (9/2020), and Turku University Hospital (PIC-99396608).

## 3 Results

### 3.1 User dimension

#### 3.1.1 Demographic, personal traits and mental health status influences

**Fig. 2:**
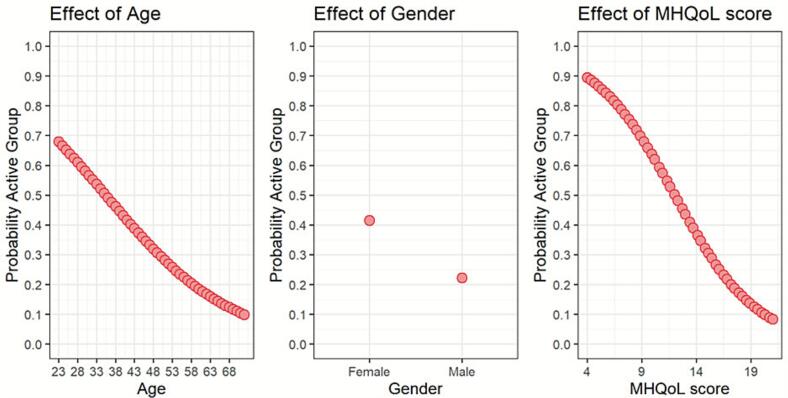
Plots of the factors that significantly influence the active or inactive use.

The results of the logistic regression analysis examining the factors predicting active versus inactive use are presented in Table 1. Factors with a significant *p*-value are presented in Fig. 2. Three variables were found to be significant: Age, Gender and mental QoL. As shown in Fig. 2, older age, poorer mental QoL and being male were associated with a lower likelihood of being an active EMPOWER application user. Qualitative findings from the interviews further supported the influence of demographic and contextual factors, such as gender, age, and family circumstances, on the amount of application usage. As suggested by the employers: “*Particularly in older people, that they just do not want it, it seems too burdensome, and they would rather do it in some other way” (Employer 1, F)*. Other general characteristics that could influence outcomes according to the employers were: kind of work, education, and job position.

**Table 1:** Output of the logistic regression model in which factors are identified that may potentially influence the active versus inactive use.

Term	Odds ratio^1^
Age	0.942 (0.91, 0.976)***
Gender	
Female	Reference
Male	0.411 (0.192, 0.879)*
Education	
Primary school	9.028 (0, >100)^2^
Secondary school	0.000 (0, >100)^2^
GCSE	64.028 (0, >100)^2^
Bachelor's degree	9.964 (0, >100)^2^
Doctoral degree	Ref (sum to zero baseline)
Company size	1.000 (0.999, 1)
PHQ9	0.919 (0.84, 1.005)
MHQoL	0.764 (0.647, 0.901)
MHQoL – VAS	1.291 (0.994, 1.677)
Sitting	1.001 (1, 1.002)
iPCQ – Presenteeism	1.022 (0.997, 1.048)
Marital status	
Cohabitating	0.054 (0, >100)^2^
In a relationship	0.022 (0, >100)^2^
Married	0.068 (0, >100)^2^
Separated/divorced	0.151 (0, >100)^2^
Single	0.087 (0, >100)^2^
Widowed	Ref (sum to zero baseline)

*P-value < 0.05.

**P-value < 0.01.

***P-value < 0.001.

1 Odds Ratio (95% Confidence Interval).

2 Wide intervals (0, >100) indicate estimation instability due to sparse data.

In contrast to the findings above, the linear regression (Appendix B, Table 1) did not identify significant associations between higher age, male gender and higher mental QoL with the amount of application starts. However, marital status emerged as a significant factor, with individuals in a relationship demonstrating notably lower levels of application starts.

#### 3.1.2 Perceptions of mental health and digital health interventions

Employees' perceptions were reflected in their reasons for joining the EMPOWER trial. The most common was an interest in monitoring and improving wellbeing or coping with mental health challenges, showing the importance placed on self-management. Others expressed a more general curiosity about mental health, seeking to broaden their knowledge: *“I've always been very much interested in mental health issues anyway, so I like to get involved in things like that.” (Employee 10, F, f, 27).* A smaller group participated out of interest in research participation itself.

#### 3.1.3 Prior experience with (mental) health technology and skills

Few employees had previously used a mental health app, but prior experiences with other apps shaped expectations. Three features were highlighted as added value of EMPOWER. First its workplace orientation made it feel more relevant to employees' needs. Second, it was perceived as credible and research-based: *“might be more of a source of researched information than these commercial services” (Employee 9, F, m, 40)*. Third, it offered broader content, with more emphasis on mental health compared to apps focusing mainly on physical wellbeing: *“Quite often, even in health centres, when we talk about self-care, it is very much focused on the physical view of a person. One should always try to remember the psychophysical-social whole of a person, that it has all three different sides and take care of them all” (Employee 12, F, f, 34).* Overall, employees expected EMPOWER to be trustworthy, holistic, and suitable for the work context, and felt confident in their ability to use it without further support.

#### 3.1.4 Integration of digital health into life

Although designed for workplace use, employees mostly engaged at home due to lack of time at work: *“Almost impossible at work, we have such a tight schedule” (Employee 12, F, f, 34).* As a result, respondents reported a decrease in the frequency of app usage over time, particularly as the study progressed. One respondent noted: *“Let's say that in the beginning I used it maybe daily and then towards the end less often.” (Employee 1, F, f, 48).* Most employees engaged with the intervention at their own pace, adapting usage to their personal schedules.

Another factor influencing integration was uncertainty regarding the provided instructions. Employees often did not read or follow the guidance, which contributed to inconsistent usage patterns. As one employee stated: *“Let's put it this way, I just started thinking about what the instructions were. I can't remember any of them” (Employee 1, F, f, 48).*

### 3.2 Technology and environment

#### 3.2.1 Technology-related factors

Overall usability was rated as good, with employees finding the app easy to navigate and appreciating the convenience of mobile access. However, the repeated log-in requirement after inactivity was a common frustration, creating a barrier to quick use “…*that you have to log in every time…, it's a bit miserable.” (Employee 7, F, f, 62).*

#### 3.2.2 Privacy and confidentiality

Among Polish respondents, privacy concerns emerged as a major barrier to uptake and use of the EMPOWER platform, particularly regarding the personal data required at installation: “…*some kind of a barrier may be personal data that is required during its installation”(Employer 10, P).* Employees in Poland feared data misuse, with worries shaped by broader issues such as online scams, breaches, and the perception that workplace apps could be used for monitoring. The hesitation was reflected in employees' accounts of being asked to provide identifying information during installation, as one employee explained: *“If I remember correctly, it asked for the ID card number, and I must admit honestly, it worried me a bit, because for a user unfamiliar with NIOM and without knowing who is behind it, I would not use such an application anymore” (Employee 24, P, f, 23)* . Despite assurances of anonymity, these concerns persisted among Polish participants, suggesting that they were rooted in broader cultural and social dynamics rather than in the design of the EMPOWER platform itself. Importantly, privacy-related concerns were not reported by participants in Spain or Finland, indicating clear contextual differences in perceptions of data security across countries.

#### 3.2.3 Social influence

Workplace attitudes towards mental health varied by country, reflecting differences in country and workplace culture and attitudes. In Finland, attention to mental health was seen as dependent on supervisors' views rather than corporate culture. Many employees observed that attitudes towards mental health were shaped primarily by the personal approach of individual supervisors and the atmosphere they created, rather than by organisational policies, as one employee stated: *It's more in the hands of the supervisor and the atmosphere that they create.” (Employee 19, F, m, 47)*. Many employees felt that too little or no attention was paid to mental healthcare in their workplace, indicating a lack of systemic focus. In contrast, employees in Poland believed that sufficient attention was given to mental health in their organisations. However, specific examples of this perceived attentiveness were lacking, and comments were often vague, such as: *“The atmosphere is such that I think that if something happened, the institute would pay attention.” (Employee 21, P, m, 28).* In Spain, employee opinions were divided. Half of the respondents believed that their workplaces provided adequate attention to mental health issues, while the other half felt that such attention was either minimal or lacking.

Among employers, opinions on how mental health was addressed in the workplace were similarly divided. Some employers indicated that their organisations had established structured processes for managing mental health issues, such as early intervention models: *“So we have an early intervention model and concern discussion” (Employer 1, F).* In contrast, others noted that little or no attention was paid to mental health problems in their organisations emphasizing a reactive rather than proactive approach. One employer remarked: “*I don't have any experience. Because no one reports such problems, or at least it seems to me that no one at the Institute reports such problems to the supervisor. And if someone has mental health problems, he goes to his doctor and so, probably the supervisor is the last person who even knows about such problems.” (Employer 11, P).*

Employees did not perceive any changes in workplace attitudes towards mental health as a result of the platform's implementation. In follow-up interviews, they reported that the intervention did not lead to additional conversations or initiatives related to mental health. One employee commented: “*In fact, I haven't even asked my team if they've implemented this … So, I don't recall any story about this that anyone has spoken out loud. So, I don't even know from my own team members how many people were involved in this.” (Employee 13, F, f, 45).*

Similarly, employers observed little to no impact of the platform on how mental health issues were addressed in their organisations. One employer summarized the lack of change, stating: *There's nothing, just the same. There's no change that way. There's no change, no change.” (Employer 2, F).*

Overall, while the platform may have had the potential to influence workplace conversations around mental health, its impact on social influence within organisations was minimal and appeared to depend heavily on the cultural context and the social structures already established within each organisation.

#### 3.2.4 Implementation

Employers highlighted communication as critical for successful implementation. Clear information on availability and purposed was seen essential to engage employees, even those without current health problems *“it requires pretty good communication at least (Employer 2, F),* suggesting that employees might not immediately recognize the relevance of the platform unless it is actively brought to their attention.

Beyond raising awareness, some employers highlighted the need to actively convince employees of the platform's benefits. One suggested that an information campaign would be valuable, as employees are more likely to engage when they understand how it can positively impact their well-being, as one employer explained:

*“I think an information campaign is important. Employees need to know there is a possibility to use such application. Then I think that if they are convinced that it can benefit their health, it will bring some benefits for them personally, I think that it will help to take this action and act.” (Employer 12, P).*

To improve implementation, others stressed the importance of maintaining a steady flow of information over time, combined with clear guidance both within and outside the application. One employer pointed out that direct outreach from experts would likely be more effective than relying on line managers, explaining that: *“That marketing, (…) it makes sense to do it in a different way, and more closely with experts directly reaching out to those employees, rather than through a line manager on the sidelines of this project.” (Employer 3, F*).

Others also emphasised the need for tailored communication strategies that resonate with employees. Some proposed using internal social media campaigns to make information more accessible and engaging. Recognizing the challenge of reaching employees effectively, one employer suggested spreading the message through different channels, remarking: *“I mean, you can try to reach through, I don't know, some campaigns on social networks. Well, I also know how it goes, because I have such a problem too. You just have to spread it out so that it's for these people.” (Employer 13, P).*

Despite their emphasis on communication as a critical factor for implementation, employers generally did not see responsibility for communication as their role. This perception left communication efforts unresolved, as no single entity took ownership of ensuring that employees received consistent information about the platform. Instead, employers suggested that responsibility should lie elsewhere, with more structured and targeted communication strategies provided by external experts. As one employer explained: *“That marketing, which is what I was talking about. It makes sense to do it in a different way, and more closely with experts directly reaching out to those employees, rather than through a line manager on the sidelines of this project.” (Employer 3, F).* As a result, the boundary between aspects related to the research study and those directly connect to the platform was unclear for both employers and employees. This lack of clarity caused confusion, as users often struggled to distinguish whether certain features or requirements (such as the lengthy questionnaires) were part of the research or integral to the platform itself. Hence, this overlap blurred the lines between the research process and the functionality of the platform. Which led for many users to an overall ambiguous perception of the platform, making it harder for them to engage with the application and fully understand its intended purpose. As one employee explained: *“But it was kind of confusing, because I thought at first that once I had filled in the questionnaire, I would be able to get to know the application, but that didn't happen. And I thought at first that it somehow does not work.” (Employee 2, F, f, 46).*

### 3.3 Program dimension

#### 3.3.1 Perceived fit, effectiveness and usefulness

At baseline, employees expected the EMPOWER platform to deliver three key benefits: improved knowledge and insight in their own health, skills to cope with mental health problems, and a reduced stigma around discussing mental health in the workplace. Among employees who experienced an effect after using the platform, the most commonly reported impact was increased personal awareness:

*“Actually, it's a permanent change in mindset. It's funny that an app can make you think this way, but it's understandable to have feelings of inadequacy, just like others do, and it's completely normal. Even though we talk about it all the time, somehow, it still works when it reminds you of its existence.” (Employee 5, F, f, 44).*

Employers shared similar optimism about the platform's potential, suggesting it could encourage employees to reflect on their mental health and increase awareness of mental health issues:

*“It's looking in the mirror and generally stopping to think about some of the things that they're either not aware of, or unconsciously or consciously sweeping under the carpet. When those questions come up, they force you to think about your own mental health and how you can influence it.” (Employer 3, F).*

Nevertheless, not all employees reported noticeable effects. Despite this, they remained positive about the platform's design and functionality. Many noted their satisfaction with its readability, variety and simplicity: *“I liked it yes yes … There is a lot of information but it is very enjoyable to read’ (Employee 31, S, f, 42)*; ‘*It was a positive surprise that it was such a varied program’ (Employee 5, F, f, 44);* and *“I think that is quite useful and its simplicity helps a lot” (Employee 33, S, m, 36).*

While employees were generally positive about the platform, several barriers emerged that impacted their motivation and usage. One commonly reported issue was the length of the research-related questionnaires, which many participants found too long and time-consuming. This was frequently mentioned in post-intervention interviews, with some employees describing the survey as demotivating. One participant stated:*”I was discouraged by the fact that I was completing the first, very long questionnaire, and I received nothing in the sense of such feedback.” (Employee 23, P, f, 24).*

Regarding program usage within the application, onboarding and mood tracking were the only modules used by more than 70% of employees in both the active an inactive groups (Appendix A, Table 4). In the active group, additional modules such as book readings and habit creation/tracking were also used by around 50% or more of employees. Other programs, however, were utilized less frequently. In contrast, employees in the inactive group rarely used additional modules. Interviews provided insight into the variation in program usage, indicating that the perceived usefulness of features was highly dependent on the individual circumstances of the respondents. Employees' preferences for specific programs varied significantly, reflecting the importance of tailoring interventions to their unique needs. Hence, personalised programs, such as mood tracking, were consistently regarded as more engaging and beneficial.

Participants emphasised the value of tailored features, suggesting that programs offering personalised feedback would improve user engagement. As one employee noted: “*for people to feel that they get some clear benefit from giving information to an application …that makes it visible that it gives me tailored information” (Employee 2, F, f, 46)*. These findings highlight the importance of incorporating personalisation into platform design to better meet the diverse needs of users and enhance the overall user experience.

#### 3.3.2 Guidance on the usage of the intervention

Guidance on platform use was minimal. Beyond the initial invitation email, no further communication was provided (by either employers or the research team), as employees reported that additional guidance was unnecessary. Most found the in-app information easy to following and felt confident using the platform independently. However, some uncertainty arose in the early stages. A few participants described difficulties locating or accessing the app:

*“Yeah, well, at the beginning I have to say it was a bit confusing, getting there in the first place. They talked about the app in the email, and then there was a QR code to get there, but I didn't find the actual app in Apple's AppStore.” (Employee 9, F, m, 40).*

#### 3.3.3 Ability of the intervention to create social connections and positive impacts

For most employees, the platform raised awareness of mental health but did not stimulate broader discussion or social connection: “…*I do not feel the subject [of mental health] is discussed more often.” (Employee 32, S, f, 27).* Employers, however, viewed the intervention as a potential catalyst for cultural change. They believed it could normalise conversations and lower the threshold for seeking help: *“This could perhaps bring awareness, and show that even small things are mental health issues, and sleeping issues and others, so it could lower that threshold, and it could perhaps be easier to talk about these.” (Employer 2, F).* Others emphasized its role in encouraging proactive action: “…*if I am aware of what may happen to me or what I am struggling with, I will act against it” (Employer 14, P)*. This highlights a discrepancy between employers' expectations of workplace impact and employees' largely individual experiences.

## 4 Discussion

### 4.1 Reflection on main findings

**Fig. 3:**
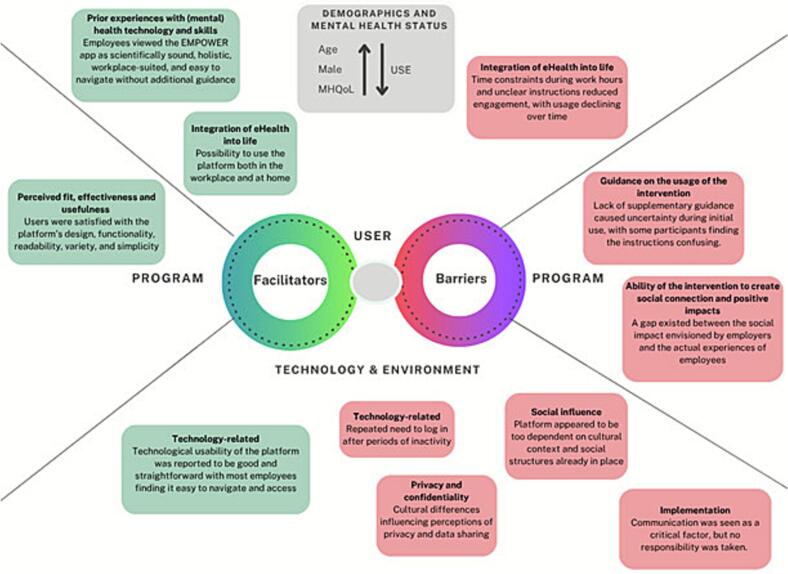
Perceived barriers and facilitators associated with the uptake of the EMPOWER study.

This study examined engagement with the EMPOWER platform in workplace settings, identifying barriers and facilitators to its use through a mixed-methods analysis guided by the categorisation framework of Borghouts et al. (Borghouts et al., 2021). By integrating qualitative insights from employees and employers with quantitative analyses of baseline characteristics associated with platform use, this study provides a comprehensive understanding of how individual, organisational, and contextual factors shape engagement with a digital mental health intervention. Fig. 3 summarises the key facilitators and barriers identified.

Overall, the findings demonstrate that engagement with the EMPOWER platform was strongly shaped by organisational conditions within participating workplaces. Although prior research consistently highlights the importance of employer engagement, supportive workplace cultures, and clear communication for the successful implementation of workplace digital health interventions (Cremers et al., 2021; Bente et al., 2024; Taylor et al., 2015; Ross et al., 2016; Kruszyńska-Fischbach et al., 2022), these conditions were not consistently present across the organisations participating in this study. The qualitative findings revealed several prominent organisational barriers to uptake, including insufficient communication and guidance, uncertainty during initial use, lack of clearly allocated time during work hours and a misalignment between employer's expectations of social or cultural impact and employees' everyday experiences. Employees frequently reported confusion regarding how and when to use the platform, ambiguity surrounding questionnaires, limited practical guidance, and concerns about anonymity. These challenges were closely tied to stakeholder dynamics within organisations. Employers often expressed reluctance to take responsibility for communication and facilitation, perceiving mental health support as a sensitive domain or viewing themselves as an inappropriate intermediary. The lack of clear ownership contributed to fragmented implementation processes and reduced engagement.

One explanation of these challenges lies in the dual nature of the EMPOWER project, which functioned both as a workplace intervention and as a research study requiring data collection. This dual role appeared to create uncertainty among employees, particularly regarding the necessity and relevance of questionnaires that would not typically be part of a routine workplace initiative. The triangular communication structure involving employers, researchers, and employees likely contributed to a diffusion of responsibility, leaving gaps in communication and workplace-based support insufficiently addressed. Consequently, no measurable changes in mental health awareness or workplace mental health culture were observed, even though employers acknowledged the platform's potential to contribute to such outcomes. A second explanatory factor concerns organisational readiness. Previous research has emphasised that a workplace culture characterised by mutual trust, both between employees and employers and in the digital intervention itself, is critical for successful uptake of workplace mental health (Kruszyńska-Fischbach et al., 2022; Touré et al., 2012). In the present study, such trust was not consistently evident, particularly with respect to data privacy and the perceived intentions behind the intervention. In settings where trust and organisational support were limited, employees appeared hesitant to engage with a platform addressing sensitive mental health topics.

At the individual level, quantitative analyses of baseline data showed that higher baseline mental quality of life, older age, and male gender were associated with lower engagement with the EMPOWER platform. These patterns suggest that perceived need and relevance play an important role in motivating use. This interpretation is consistent with the Andersen Behavioral Model of Health Services Use, which posits that perceived need is a central determinant of health service utilisation and that individuals are less likely to seek or engage with services when they do not perceive themselves as having a health problem or risk (Andersen, 1995). In the context of a preventive digital mental health intervention, employees reporting better baseline mental health may therefore experience a lower perceived need, reducing their motivation to engage. Lower engagement among older employees and men has been discussed in previous research on digital health and mental health interventions, however, such patterns have predominantly been identified through qualitative or descriptive studies, rather than on quantitative associations (Chen et al., 2023; Szinay et al., 2023). The present study extends this literature by demonstrating these associations quantitatively, while also indicating that individual characteristics alone were insufficient to explain uptake in the absence of supportive organisational conditions. In addition, cultural differences in perceptions of trust, privacy, and relevance emerged from the qualitative analyses, highlighting how contextual factors shape engagement in ways that are not readily captured through quantitative measures (Boucher and Raiker, 2024; Naderbagi et al., 2024). The inclusion of multiple countries added important contextual insights: while cross-country comparisons were not the primary focus of the analysis, qualitative data from Spain, Finland, and Poland revealed meaningful differences in how the EMPOWER platform was interpreted and experienced particularly with regard to privacy concerns, trust in data handling, and the perceived role of employers. These findings underscore that cultural context influences engagement with workplace digital mental health interventions. Although these patterns were explored descriptively rather than comparatively, they point to the need for future research to more explicitly integrate cultural and contextual dimensions into implementation frameworks.

The categorisation framework proposed by Borghouts et al. proved useful for structuring and interpreting the findings across both qualitative and quantitative components of this study (Borghouts et al., 2021). Most identified barriers and facilitators could be meaningfully mapped onto the user, technology and environment, and program dimensions. However, the findings also indicated that some experiences did not fit within a single dimension, with several factors, such as communication, trust, and perceived responsibility, cutting across multiple domains. This overlap reflects the complex and interdependent nature of implementing digital mental health interventions in non-healthcare workplace settings, for which the framework was not originally developed. Moreover, the study highlighted the relevance of an additional research-related dimension that is not explicitly captured with the Borghouts et al. (Borghouts et al., 2021) framework. Research-related elements, including data collection procedures, questionnaires, and trial-related communication, substantially influenced engagement and trust. These aspects are often underrepresented in implementation frameworks, yet they play a critical role in shaping uptake in real-world trial contexts.

### 4.2 Strengths and limitations

One of the strengths of this study was the involvement of multiple countries, allowing comparisons of multiple (organisational) cultures. Secondly, the interviews were conducted both with employers and employees, which made it possible to present the facilitators and barriers from multiple viewpoints. Thirdly, both qualitative and quantitative techniques were used, enabling the implementation of cross-validation. When focusing on workplace-specific digital health interventions, organisational culture and stakeholder involvement are consistently identified as important factors for successful implementation and uptake (Bernard et al., 2022; Nöhammer and Drexel, 2024) . This study, however, stands out by identifying specific demographic factors that either increase or decrease the likelihood of using the intervention. Additionally, this study considers the influence of the research setting, a factor not addressed in other studies. In contrast, aspects such as supportive legislation, adherence to recognized standards, national culture, and other external condition beyond the workplace environment, were not included in this study. As highlighted in previous research, these factors can also significantly impact the uptake process within the workplace setting (Bente et al., 2024; Ross et al., 2016; Kruszyńska-Fischbach et al., 2022). Thereby, there may be a bias in the sample, as the group participating in the study already had positive attitudes towards the subject of mental health. Furthermore, those who agreed to be interviewed may have been even more positive and more interested in the EMPOWER application. In addition, the number of participating employers for the qualitative analyses was low at both baseline and post-intervention, which may have led to important facilitators and barriers being overlooked. The same limitation applies to the post-intervention interviews conducted with employees. Furthermore, the number of respondents per country varied, as did the length of the interviews. Hence, important facilitators and barriers could have been missed. Due to resource constraints in the EMPOWER study, participants from the UK were only involved in the questionnaires, not the interviews, further impacting the robustness of the data and its implications for generalizability. Another limitation concerns the study design. Only group B participants were interviewed, primarily because it was more practical to schedule baseline interviews with them. By the time of the interviews, these respondents already completed several questionnaires for the RCT, which may have influenced their experiences of both the RCT and the EMPOWER intervention. Including group A in the qualitative research sample could have provided other perspectives. Furthermore, the timing of this study is important to consider. The interviews took place in the first year of the RCT. During the study, changes were made in the design, and actions were taken to increase the participation. Some of these actions were related to improve the communication flow with employers/managers and employees, and included: an increased contact with manager/CEOS, dissemination within the company (offering learning pills about mental health and creation of a new poster with the app QR), to report and solve technical problems, and local teams were constantly available for those participants who needed assistance. As a result, some of the suggestions made in this study were already incorporated into the new trial design. However, since the interviews were conducted beforehand, these changes were not accounted for, and their effects were not evaluated in this study. Additionally, the influence of costs on the platform's ultimate adoption and use was not assessed, as the application was provided free of charge during the trial. While this allowed all participants equal access to the intervention, it leaves open the question of how potential pricing could impact future uptake and usage in real-world settings.

### 4.3 Conclusion

The uptake of the EMPOWER intervention was primarily hindered by insufficient communication and inadequate workplace support, which significantly impacted every aspect of the uptake process. These issues arose from the interplay between key stakeholders and low organisational readiness, ultimately leading to reduced engagement. Conversely, the intervention's user-friendliness positively contributed to its outcomes. To improve future uptake of digital health interventions in workplace settings, it is essential to prioritise stakeholder engagement, particularly involving employers, while also addressing’ organisational readiness. Additionally, recognising differences between users and tailoring the application to their needs, as well as optimising communication and information delivery, are crucial to ensuring successful uptake.

## Funding

The research leading to these results has received funding from the European Union's Horizon 2020 Research and Innovation Programme under Grant Agreement No. 848180 and the National Health and Medical Research Council of Australia (APP1195937). B.O. is supported by the Miguel Servet (CP20/00040) contract, funded by the 10.13039/501100004587Instituto de Salud Carlos III and co-funded by the 10.13039/501100000780European Union (ERDF/ESF, “Investing in your future”.

## Declaration of competing interest

The authors declare the following financial interests/personal relationships which may be considered as potential competing interests: Stijn B. Peeters reports financial support was provided by European Union. Marleen de Mul reports financial support was provided by European Union. Marjo Sinokki reports financial support was provided by European Union. Kaja Staszewska6 reports financial support was provided by European Union. Luis Salvador-Carulla reports financial support was provided by National Health and Medical Research Council. Sue Lukersmith reports financial support was provided by European Union. Beatriz Olaya reports financial support was provided by Carlos III Health Institute. Beatriz Olaya reports financial support was provided by European Union. Leona Hakkaart-van Roijen reports financial support was provided by European Union. If there are other authors, they declare that they have no known competing financial interests or personal relationships that could have appeared to influence the work reported in this paper.

## Data Availability

The data that support the findings of this study are available on request from the corresponding author. The data are not publicly available due to privacy or ethical restrictions.

## Appendix A Supplementary data

Supplementary material Image 1
