# Supplementary material for: Uptake and engagement with digital mental health in the workplace: A mixed-methods analysis of the EMPOWER trial
Source: Internet Interv. 2026 Jan 20;43:100911. doi: 10.1016/j.invent.2026.100911 (PMC12861261; doi:10.1016/j.invent.2026.100911)
Supplement: Supplementary file 1 — Supplementary material [file mmc1.docx]

# **APPENDIX A – DESCRIPTIVE TABLES**

Table 1: General characteristics of the employees participating in the qualitative interviews

|  |  | T0: Baseline, N = 40 | T1: Baseline, N = 14 |
| --- | --- | --- | --- |
| Age, mean years (SD) |  | 43 (10) | 45 (11) |
| Gender | | | |
| Female, n (%) | | 31 (77.5) | 12 (85.7) |
| Male, n (%) | | 9 (22.5) | 2 (14.3) |
| Education | | | |
| Primary school, n(%) | | 1 (2.5) | 1 (7.1) |
| Secondary school, n (%) | | 1 (2.5) | 0 (0) |
| Upper secondary education / post-secondary vocational education, n (%) | | 9 (22.5) | 5 (35.7) |
| Bachelor’s degree, n (%) | | 10 (25) | 4 (28.6) |
| Doctoral degree, n (%) | | 2 (5) | 0 (0) |
| Missing, n (%) | | 1 (2.5) | 1 (7.1) |
| Company type | | | |
| Public agency, n (%) | | 33 (82.5) | 8 (57.1) |
| SME, n (%) | | 7 (17.5) | 6 (42.9) |
| Country | | | |
| Finland, n (%) | | 20 (50)  Respondents: F1 – F20 | 8 (57.1) |
| Poland, n (%) | | 8 (20)  Respondents: P1-P8 | 2 (14.3)  Respondents, P9, P10 |
| Spain, n (%) | | 12 (30)  Respondents S1-S7,  S9, S10, S13-S15 | 24 (28.6)  Respondents, S1, S3, S7, S10 |

Table 2: General characteristics of the employees participating in the quantitative questionnaires, divided by active and inactive users

|  |  | Active, N = 90 | Inactive, N = 149 | Overall, N = 239 |
| --- | --- | --- | --- | --- |
| Age, mean years (SD) |  | 44 (10) | 45 (11) | 45 (11) |
| Gender | | | | |
| Female, n (%) | | 74 (82) | 110 (73) | 184 (76) |
| Male, n (%) | | 16 (18) | 41 (27) | 57 (24) |
| Education | | | | |
| Primary school completed, n (%) | | 0 (0) | 0 (0) | 0 (0) |
| Some secondary school – not completed, n (%) | | 0 (0) | 1 (0.7) | 1 (0.4) |
| Secondary school - vocational, n (%) | | 13 (14) | 9 (6.0) | 22 (9.1) |
| GCSE, n (%) | | 19 (21) | 33 (22) | 52 (22) |
| A-levels, n (%) | | 18 (20) | 18 (12) | 36 (15) |
| University degree completed, n (%) | | 40 (44) | 90 (60) | 130 (54) |
| Company type | | | | |
| Public agency, n (%) | | 68 (76) | 121 (80) | 189 (78) |
| SME, n (%) | | 22 (24) | 30 (20) | 52 (22) |
| Country | | | | |
| Finland, n (%) | | 26 (29) | 22 (15) | 48 (20) |
| Poland, n (%) | | 3 (3.3) | 4 (2.6) | 7 (2.9) |
| Spain, n (%) | | 23 (26) | 35 (23) | 58 (24) |
| United Kingdom, n (%) | | 38 (42) | 90 (60) | 126 (53) |
| MHQoL score, mean (SD) | | 13.8 (3.2) | 14.3 (3.0) | 14.1 (3.1) |
| EQ5D score, mean (SD) | | 7.36 (2.19) | 7.08 (2.20) | 7.18 (2.20) |

Table 3: General characteristics of the employers participating in the qualitative interviews

|  |  | T0: Baseline, N = 40 | T1: Baseline, N = 14 |
| --- | --- | --- | --- |
| Company type | | | |
| Public agency, n (%) | | 9 (75) | 5 (55.6) |
| SME, n (%) | | 3 (25) | 4 (44.4) |
| Country | | | |
| Finland, n (%) | | 4 (33.3)  Respondents: F1 – F4 | 4 (44.4)  Respondents: F1-F4 |
| Poland, n (%) | | 5 (41.7)  Respondents: P1-P5 | 3 (33.3)  Respondents: P1-P3 |
| Spain, n (%) | | 3 (25)  Respondents S1-S3 | 2 (22.2)  Respondents: S2 & S3 |
| Role of the employers | | | |
| Directors, n (%) | | 3 (25)  Respondents: F1, P3, S1 | 1 (11)  Respondents: F1 |
| Manager, n (%) | | 4 (33.3)  Respondents: F2, F4, P1, P2 | 4 (44.4)  Respondents: F2, F4, P1, P2 |
| Principal, n (%) | | 4 (33.3)  Respondents: F3, F5, S2, S4 | 4 (44.4)  Respondents: F3, P3, S2, S3 |
| Missing | | 1 (8.3)  Respondent: P4 | 0 (0) |

Table 4: Number of App Launches and Modules Completed, Categorized by Active, Inactive Groups, and Overall

|  |  | Active, N = 90 | Inactive, N = 149 | Overall, N = 239 |
| --- | --- | --- | --- | --- |
| Number of App launches | | | | |
| Mean (SD) | | 13 (17) | 3 (4) | 6 (12) |
| Median (IQR) | | 6 (2-14) | 2 (1-3) | 2 (1-5) |
| Programs Completed, n (%) | | | | |
| Book reading | | 62 (68.9) | 0 (0) | 62 (25.9) |
| Breathing exercise | | 32 (35.6) | 12 (8.1) | 44 (18.4) |
| Habit created | | 52 (57.8) | 9 (6.0) | 61 (25.5) |
| Onboarding | | 74 (82.2) | 113 (75.8) | 187 (78.2) |
| Problem created | | 24 (26.7) | 1 (0.7) | 25 (10.5) |
| Relaxation - Short | | 25 (27.8) | 11 (7.3) | 36 (15.1) |
| Relaxation - Long | | 5 (5.6) | 1 (0.7) | 6 (2.5) |
| Tracking - habits | | 43 (47.8) | 9 (6.0) | 52 (21.8) |
| Tracking - mood | | 87 (96.7) | 137 (91.9) | 224 (93.7) |

## **APPENDIX B – LINEAR REGRESSION MODEL**

| Term |  | Coefficient^1^ |
| --- | --- | --- |
| Intercept |  | 24.583 (8.960)** |
| Age | | -0.122 (0.083) |
| Gender | | |
| Female | | Reference |
| Male | | -3.519 (1.876) |
| Education | | |
| Primary school | | -2.272 (9.378) |
| Secondary school | | 5.618 (3.142) |
| GCSE | | -1.624 (2.777) |
| Bachelor’s degree | | 2.236 (2.927) |
| Doctoral degree (sum to zero baseline) | | -3.958 |
| Company size | | -0.0006 (0.001) |
| PHQ9 | | -0.327 (0.220) |
| MHQoL | | -0.527 (0.408) |
| MHQoL - VAS | | 0.058 (0.657) |
| Sitting | | 0.003 (0.003) |
| iPCQ - Presenteeism | | 0.088 (0.067) |
| Marital status | | |
| Cohabitating | | -4.441 (2.281) |
| In a relationship | | -7.614 (2.374)** |
| Married | | -1.086 (1.832) |
| Separated/divorced | | 0.723 (2.822) |
| Single | | -3.452 (2.710) |
| Widowed (sum to zero baseline) | | 15.87 |
| *^1^Coefficient (Standard Error)*  *^*^P-value < 0.05*  ***P-value < 0.01*  ****P-value <0.001* | |  |

## **APPENDIX C – INFORMATION ABOUT THE DIVISION OF PARTICIPANTS IN ACTIVE AND INACTIVE USERS**

Participants were classified as either active or inactive users based on their engagement with the app. Engagement was measured using the following criteria:

1. Obtaining at least one badge (a badge is further defined in the forthcoming effectiveness study)
2. Earning more than five badges
3. Reading more than one page
4. Starting at least one problem
5. Tracking mood for more than seven days

Each criterion met contributed one usage point. Users with a total of one point or fewer were categorized as inactive, while those with more than one point were classified as active.
